# Dynamic Changes in Antithyroperoxidase and Antithyroglobulin Antibodies Suggest an Increased Risk for Abnormal Thyrotropin Levels

**DOI:** 10.3389/fendo.2020.00521

**Published:** 2020-08-04

**Authors:** Yongze Li, Di Teng, Haixia Guan, Yushu Li, Xiaochun Teng, Xiaohui Yu, Jinyuan Mao, Xiaoguang Shi, Chenling Fan, Zhongyan Shan, Weiping Teng

**Affiliations:** Department of Endocrinology and Metabolism, The Institute of Endocrinology, The First Hospital of China Medical University, Shenyang, China

**Keywords:** thyroid antibodies, TPOAb, TgAb, TSH, epidemiology

## Abstract

**Background:** Antithyroperoxidase (TPOAb) and antithyroglobulin (TgAb) antibodies are associated with abnormal thyrotropin (TSH) levels. However, the effect of dynamic changes in TPOAb and TgAb on incident abnormal TSH is unknown.

**Methods:** A total of 2,387 euthyroid participants aged 18 years or older from three rural areas in northern China were enrolled in this cohort study. Questionnaire interviews and laboratory measurements were performed at baseline in 1999 and at follow-up in 2004. Multinomial logistic regression was used to examine the relationship between changes in thyroid antibodies and incident abnormal TSH levels.

**Results:** In this 5 year follow-up study, TPOAb tier gain was significantly associated with an increased risk of subnormal TSH levels (adjusted RR, 1.535; 95% CI: 1.357–1.736) and supranormal TSH levels (adjusted RR, 1.378; 95% CI: 1.196–1.587), and TgAb tier gain was significantly associated with an increased risk of supranormal (adjusted RR, 1.090; 95% CI: 1.007–1.179) TSH levels. Both thyroid antibody-positive seroconversion and persistent positivity were significantly associated with an increased risk of incident abnormal TSH levels. Thyroid antibody positive seroconversion was associated with a higher risk of incident subnormal TSH than incident supranormal TSH, whereas persistent positive thyroid antibody was associated with a higher risk of incident supranormal TSH than incident subnormal TSH.

**Conclusions:** Dynamic thyroid antibody changes may be related to incident abnormal TSH levels. Those with persistent positive thyroid antibody were more likely to have supranormal TSH than subnormal TSH, and those with positive seroconversion were more likely to have subnormal TSH than supranormal TSH. Further studies are needed to confirm this conclusion and to explore this association mediated by TSH receptor antibodies.

## Introduction

Serum thyrotropin (TSH) levels outside the reference range are common in clinical practice, particularly in elderly and pregnant women (1). Abnormal TSH levels are associated with endocrine and metabolic disorders. Previous studies have indicated that abnormal TSH during pregnancy associated with adverse outcomes including preterm delivery, miscarriage and cognitive impairment in offspring (2). The presence of thyroid autoantibodies is a sign of thyroid autoimmunity. A positive thyroid autoantibody test often indicates a potential thyroid-related autoimmune disease, even if TSH levels are still within the normal range.

Antithyroperoxidase antibody (TPOAb) and antithyroglobulin antibody (TgAb) are important thyroid autoantibodies, commonly seen in patients with autoimmune thyroid disorders (3). Studies have demonstrated that TPOAb can induce antibody-dependent cell-mediated cytotoxicity and that TPOAb titers are associated with the severity of lymphocytic infiltration with or without hypothyroidism (4, 5). Due to the development of testing techniques, highly sensitive and specific assays for TPOAb and TgAb, either separately or combined, are available to the general population (6, 7). The National Health and Nutrition Examination Survey III (NHANESIII) study reported that more than 10% of disease-free populations in the United States were presented as TPOAb- or TgAb-positive (8). A cross-sectional study in China indicated that the prevalence rate of TPOAb and TgAb positivity were 10.19 and 9.70%, respectively (9). Several cross-sectional and cohort studies have explored the relationship between thyroid antibodies and TSH (10, 11). However, previous studies were mainly based on TPOAb and/or TgAb at a certain time point and did not consider the effect of thyroid antibody changes on incident thyroid dysfunction. Whether dynamic thyroid antibody gain has an adverse effect on TSH levels is not well-documented. Therefore, we aimed to clarify the role of TPOAb and TgAb in the risk of abnormal TSH levels and to assess the relationship between dynamic thyroid antibody changes and incident abnormal TSH levels.

## Materials and Methods

### Study Design and Study Population

This community-based cohort consisted of participants ≥14 years old residing in three rural areas in northern China, as previously described (12). In brief, 3,761 participants who had been living in the selected community for at least 10 years were recruited in 1999. Questionnaire interviews and laboratory measurements were performed at baseline and at the follow-up in 2004. The follow-up population comprised 3,018 original subjects (response rate, 80.2%). For the 3,018 participants, we excluded those aged < 18 years at baseline, those with abnormal TSH levels (outside the reference range) at baseline, and those with incomplete information on age, sex, TSH, TPOAb, TgAb, and urinary iodine concentration (UIC) at both baseline and the follow-up examination. Finally, the data of 2,387 participants were collected, with a follow-up of 5 years, to assess the risk of abnormal TSH with thyroid antibody changes. The study complied with the Declaration of Helsinki and was approved by the medical ethics committee of China Medical University. All participants provided written informed consent.

### Laboratory Methods

Serum TSH, TPOAb, and TgAb levels were measured using commercial kits (Immulite 2000 chemiluminescent immunoassay; Diagnostic Products Corp., Los Angeles, CA), in the central laboratory located in Shenyang. The calibration ranges for TSH, TPOAb, and TgAb were 75 mIU/liter, 1,000 IU/ml, and 3,000 IU/ml, respectively. The analytical sensitivities of serum assays of TSH, TPOAb, and TgAb were 0.002 mIU/liter, 7 IU/ml, and 10 IU/ml, respectively. The intra-assay coefficients of variation (CV) of serum assays of TSH, TPOAb, and TgAb were 1.23–1.38, 3.51–4.65, and 3.86–6.06%, respectively. The inter-assay CV of serum assays of TSH, TPOAb, and TgAb were 1.57–4.93, 6.22–8.29, and 5.82–8.78%, respectively. The baseline and the follow-up samples were measured under the same assay methods, laboratory, commercial assay kits, and technicians in 1999 and 2004, respectively. UICs were determined using the colorimetric ceric ion-arsenious acid ash method as described previously (13). The intra- and interassay CVs for the UICs were <6.7%.

### Reference Ranges

We determined the reference range of serum TSH according to the NACB guidelines, which the range was derived from the 2.5th to 97.5th percentile of TSH (log-transformed) of subjects in this survey without thyroid antibodies, without goiter or nodules on B-mode ultrasonography, and without a known personal or family history of thyroid disease (12). Euthyroidism was defined as a TSH level between 0.3 and 4.8 mIU/liter, Although the reference ranges of TPOAb and TgAb provided by the test kit manufacturer were 35 and 40 IU/ml, respectively, we determined the cut-off values of TPOAb and TgAb were 50 and 40 IU/ml for TPOAb and TgAb, respectively, as previously reported (11, 12).

### Statistical Analysis

Baseline characteristics of the study population are presented as numbers (percentages) for qualitative variables, and medians (interquartile ranges, IQRs) and means (standard deviations, SDs) for quantitative variables, as appropriate. Student's *t*-test, the Mann-Whitney U test or the chi-square test was used to test differences between groups. Multinomial logistic regression models were used to compute the risk ratios (RRs) and 95% confidence intervals (CIs) for analyzing the risk of abnormal TSH with changes in thyroid antibodies at follow-up. The model was adjusted for age, sex, UIC and TSH at baseline. All analyses were conducted with SAS 9.4 (SAS Inst. Inc., Cary, NC, USA), and all reported *p*-values were two-sided, with *p* < 0.05 considered statistically significant.

## Results

### Sample Characteristics

The overall study population at baseline consisted of 2,387 participants (547 men and 1,840 women) who were 18–79 years old. The baseline characteristics of the study participants in the TPOAb and TgAb groups are presented in Table 1. The positive thyroid antibody participants were older, were more likely to be women and had markedly higher TSH than the negative thyroid antibody participants (all *p* < 0.05).

**Table 1:** Baseline characteristics of the study participants by TPOAb and TgAb group.

**Baseline characteristics**	**TPOAb**			**TgAb**		
	**Positive (*n =* 190)**	**Negative (*n =* 2,197)**	***p*-value**	**Positive (*n =* 203)**	**Negative (*n =* 2,184)**	***p*-value**
Age, mean (SD)	41.38 (11.51)	38.65 (11.98)	0.003	40.55 (11.23)	38.71 (12.02)	0.04
Men, *n* (%)	18 (9.47)	529 (24.08)	<0.0001	12 (5.91)	535 (24.50)	<0.0001
Women, *n* (%)	172 (90.53)	1668 (75.92)		191 (94.09)	1649 (75.50)	
TSH, mean (SD)	2.06 (1.15)	1.65 (0.94)	<0.0001	2.00 (1.11)	1.65 (0.94)	<0.0001
FT3, mean (SD)	3.11 (0.42)	3.13 (0.51)	0.7	3.11 (0.42)	3.13 (0.52)	0.7
FT4, mean (SD)	11.32 (1.16)	11.44 (1.67)	0.18	11.33 (1.29)	11.44 (1.67)	0.37
UIC, median (IQR)	327.73 (160.04–568.03)	352.68 (163.18–599.03)	0.51	345.71 (146.50–549.83)	352.40 (166.12–600.23)	0.26

### Positive Thyroid Antibodies and Incident Abnormal TSH Levels

During the 5 year follow-up, TSH<0.3 mIU/L developed in 59 of 2,387 participants, and TSH>4.8 mIU/L developed in 53 of 2,387 participants. The cumulative incidence rates of TSH<0.3 mIU/L for participants 18–39, 40–59, and ≥60 years old were 2.3, 2.8, and 2.1%, respectively. For men and women, they were 1.1 and 2.9%, respectively. The cumulative incidence rates of TSH > 4.8 mIU/L in participants 18–39, 40–59, and ≥60 years old were 2.1, 2.4 and 2.1%, respectively. For men and women, they were 2.0 and 2.3%, respectively (Table 2). The incidence of abnormal TSH levels was greater in participants with positive thyroid antibodies than in those with negative thyroid antibodies (all *p* < 0.05).

**Table 2:** Incidence of abnormal TSH levels by risk factors.

**Baseline characteristics**	**Sample size**	**TSH <0.3 mIU/L**		**TSH >4.8 mIU/L**	
		**No. of cases**	**Incidence (%)**	**No. of cases**	**Incidence (%)**
**Age (years)**					
18–39	1,334	31	2.3	28	2.1
40–59	907	25	2.8	22	2.4
≥60	146	3	2.1	3	2.1
*p* for difference			0.76		0.859
**Sex**					
Men	547	6	1.1	11	2.0
Women	1,840	53	2.9	42	2.3
*p* for difference			0.018		0.665
**TPOAb**					
Positive	190	11	5.8	15	7.9
Negative	2,197	48	2.2	38	1.7
p for difference			0.001		<0.0001
**TgAb**					
Positive	203	11	5.4	17	8.4
Negative	2,184	48	2.2	36	1.6
*p* for difference			0.002		<0.0001
**UIC (ug/L)**					
<100	356	9	2.5	6	1.7
100–199	375	7	1.9	6	1.6
200–299	316	12	3.8	5	1.6
≥300	1,340	31	2.3	36	2.7
*p* for difference			0.399		0.388

### Changes in Thyroid Antibody Tiers and Incident Abnormal TSH Levels

As shown in Table 3, the risk of TSH<0.3 mIU/L (adjusted RR = 1.535, 95% CI = 1.357–1.736) and TSH > 4.8 mIU/L (adjusted RR = 1.378, 95% CI = 1.196–1.587) increased with TPOAb tier gain among all the participants. Likewise, the risk of TSH > 4.8 mIU/L increased with TgAb tier gain (adjusted RR = 1.090, 95% CI = 1.007–1.179) among all the participants.

**Table 3:** Multinomial logistic regression analysis of thyroid antibody tier changes and the risk of abnormal TSH levels.

**Characteristics**	**TPOAb tier gain per 100 mIU/L**			**TgAb tier gain per 100 mIU/L**		
	**Risk ratio**	**95% CI**	***p*-value**	**Risk ratio**	**95% CI**	***p*-value**
**Risk of TSH <0.3 mIU/L cases**						
Crude RR (95% CI)	1.523	1.351–1.716	<0.0001	0.939	0.816–1.080	0.38
Adjusted RR (95% CI)^*^	1.535	1.357–1.736	<0.0001	0.930	0.806–1.072	0.316
**Risk of TSH >4.8 mIU/L cases**						
Crude RR (95% CI)	1.423	1.241–1.632	<0.0001	1.055	0.978–1.138	0.167
Adjusted RR (95% CI)^*^	1.378	1.196–1.587	<0.0001	1.090	1.007–1.179	0.032

* *Adjusted for age, sex, UIC, and TSH at baseline*.

### Dynamic Changes in the Status of Thyroid Antibodies and the Risk of Abnormal TSH Levels

**Figure 1:**
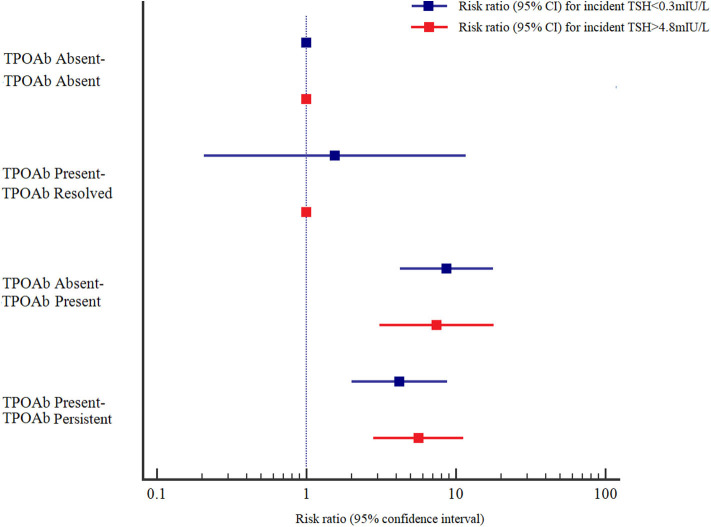
Adjusted risk ratios (RRs) and 95% confidence intervals (95% CIs) for the association of TPOAb and abnormal TSH levels by TPOAb status at baseline and at follow-up. Adjusted RRs and 95% CIs were adjusted for age, sex, UIC, Tg, and TSH at baseline.

**Figure 2:**
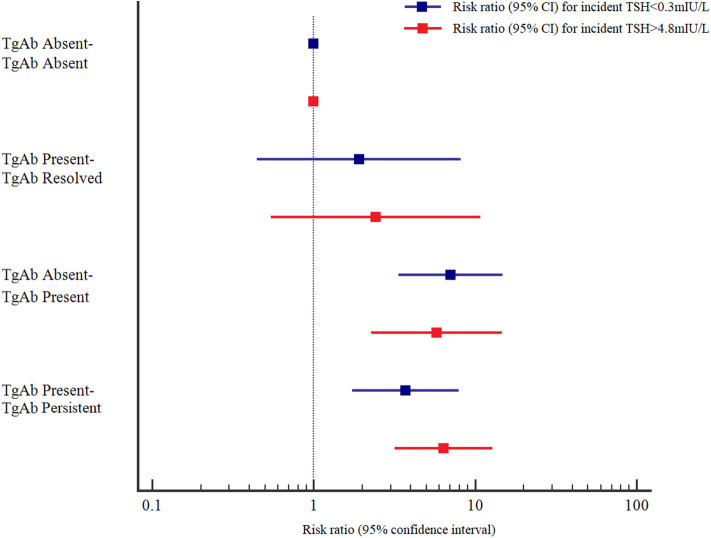
Adjusted risk ratios (RRs) and 95% confidence intervals (95% CIs) for the association of TgAb and abnormal TSH levels by TgAb status at baseline and at follow-up. Adjusted RRs and 95% CIs were adjusted for age, sex, UIC, Tg, and TSH at baseline.

The adjusted RRs (95% CIs) at baseline and follow-up in the TPOAb absent-TPOAb present and TPOAb present-TPOAb persistent groups were 8.666 (4.218–17.804) and 4.193 (2.008–8.753), respectively, for incident TSH<0.3 mIU/L, and 7.444 (3.092–17.924) and 5.617 (2.807–11.238), respectively, for incident TSH > 4.8 mIU/L compared with those in the TPOAb absent-TPOAb absent group. The adjusted RRs (95% CIs) at baseline and follow-up in the TgAb absent-TgAb persistent and TgAb present-TgAb persistent groups were 7.059 (3.363–14.815) and 3.704 (1.731–7.924), respectively, for incident TSH <0.3 mIU/L, and 5.771 (2.264–14.707) and 6.384 (3.180–12.818), respectively, for incident TSH > 4.8 mIU/L compared with those in the TgAb absent-TgAb absent group. Those with persistent positive TPOAb or positive TgAb had a higher risk for incident TSH > 4.8 mIU/L than for incident TSH < 0.3 mIU/L; however, those with positive seroconversion of TPOAb or TgAb had a higher risk for incident TSH < 0.3 mIU/L than for incident TSH > 4.8 mIU/L (Figures 1, 2).

## Discussion

In this population-based cohort study, the risk of abnormal TSH levels was increased at follow-up for euthyroid participants with positive seroconversion or persistent positive thyroid antibodies from baseline to follow-up. We found that in participants with persistent positive thyroid antibodies, the risk of developing supranormal TSH was higher than the risk of developing subnormal TSH, whereas participants with positive seroconverted thyroid antibodies were more likely to develop subnormal TSH than supranormal TSH.

Previous studies on the relationship between thyroid antibodies and TSH showed similar results (11, 13, 14). The presence of TPOAb in serum during a median follow-up of 9.1 years was reported to be associated with an increased incidence of hypothyroidism (15). In the Whickham Survey, autoimmune thyroid disorders as demonstrated by elevated TPOAb and TgAb, were associated with an increased risk of developing hypothyroidism (14). Another cross-sectional study showed that the prevalence of positive thyroid antibodies was significantly higher in participants with thyroid dysfunction than in those with euthyroidism (11). Our previous study also found that participants with positive TPOAb and TgAb at baseline had a higher risk of developing abnormal TSH than seronegative participants (10). This seems quite obvious since presumably the latter are normal subjects; On the other hand, it is well-known that up to 10–15% of patients with Hashimoto's thyroiditis (HT) may have none of these antibodies; however, the diagnosis of HT should be confirmed by ultrasound.

The polyclonality of thyroid autoantibodies indicates their variable ability to fix complement factors (primarily IgG1 and IgG3); therefore, they may contribute to thyroid damage in patients with HT. However, there is evidence that they are usually a secondary phenomenon to thyroid damage inflicted initially by T cells, thus serving as markers for detecting the disease and its severity. It is generally believed that TPOAb can bind complement factors; this plays an important role in the destruction of thyroid cells through complement-mediated cytotoxicity and is the main sign of autoimmune damage (16). The presence of TPOAb may act as a biomarker of future thyroid dysfunction (17, 18). However, the function and significance of TgAb remain controversial (8, 19). The results from the NHANES III study indicated that TgAb in the absence of TPOAb was not significantly associated with thyroid disease (8). O'Leary et al. also found that elevated TgAb values were not associated with elevated TSH levels (19). In the present study, we found that dynamic changes in both TPOAb and TgAb affected serum TSH.

Changes in serum TSH within the reference range might be correlated with slightly abnormal thyroid function of the individual, particularly if there is evidence of a thyroid-related autoimmune disease with detection of elevated thyroid antibodies (20). We hypothesized that the mechanism of dynamic status change in thyroid antibodies, inducing abnormal TSH, is similar to the phenomenon of transient thyrotoxicosis. Thyroid antibodies can destroy thyroid cells. On the one hand, large amounts of T3 and T4 stored in cells that are not initially released into the bloodstream may enter the bloodstream after cell destruction, causing decreased TSH; on the other hand, with increased cell destruction, the amounts of T3 and T4 will eventually run out, inducing elevated TSH. Therefore, positive thyroid antibodies indicate a symptom of immune dysfunction in humans. Another plausible mechanism is the potential role played by the TSHr stimulating antibody; however, we could not obtain data for thyrotrophin receptor antibody (TRAb). Thus, the mechanism of TPOAb changes should be evaluated with caution. Thyroid function maybe in the normal range during the incubation period of the disease. Regular follow-up testing should be performed, especially for pregnant women or elderly individuals. Early intervention has very important clinical significance.

To the best of our knowledge, this is the first investigation to explore the potential relationship between dynamic changes in thyroid antibodies and incident abnormal TSH levels and include standardized longitudinal assessments of thyroid function and well-measured covariates. Nevertheless, some limitations merit consideration. First, the role of TRAb is important in evaluating the impact of thyroid antibody changes on TSH levels. However, data on TRAb were not available in our study. Second, TSH levels may also be influenced by body mass index, smoking and diet, and we could not adjust for these confounders due to the study design. It is well-known that obesity may alter thyroid function in the absence of thyroid antibodies. Further studies should take into account the role of the above factors to assess the relationship between changes in thyroid antibodies and TSH levels. Although more recent studies with repeated measurements of thyroid hormone levels are needed to confirm the results in the current study, it might provide some background information regarding the reference range for TSH and a tool for physicians to predict the long-term risk of subclinical thyroid disorders in patients based on the status of thyroid antibodies.

## Conclusions

In conclusion, our results show that dynamic thyroid antibody changes may be related to incident abnormal TSH levels. Participants with persistent positive thyroid antibodies were more likely to develop supranormal TSH than subnormal TSH, and those with positive seroconversion were more likely to develop subnormal TSH than supranormal TSH. People with euthyroidism with positive thyroid antibodies should have their thyroid function monitored regularly to ensure an early diagnosis of abnormal TSH levels. Monitoring thyroid antibody changes is helpful in the primary prevention of subclinical thyroid disorders. Further studies are needed to confirm this conclusion and to explore this association, mediated by TRAb.

## Data Availability Statement

The data that support the findings of this study are available from the corresponding author upon reasonable request.

## Ethics Statement

The studies involving human participants were reviewed and approved by Medical Ethics Committee of China Medical University. The patients/participants provided their written informed consent to participate in this study. Written informed consent was obtained from the individual(s) for the publication of any potentially identifiable images or data included in this article.

## Author Contributions

WT and ZS conceived and designed the study and supervised the study. YoL did the statistical analysis. YoL, DT, HG, YuL, XT, XY, JM, XS, and CF contributed to acquisition, collection, analysis, or interpretation of data. YoL drafted and revised the manuscript. All authors approved the final version before submission.

## Conflict of Interest

The authors declare that the research was conducted in the absence of any commercial or financial relationships that could be construed as a potential conflict of interest.
